# Case Report: Cutaneous melanocytic schwannoma with concomitant melanocytoma in a canine

**DOI:** 10.12688/wellcomeopenres.19694.2

**Published:** 2024-02-26

**Authors:** Olwam H. Monakali, Nicolize O'Dell, Louise van der Weyden

**Affiliations:** 1 Department of Paraclinical Sciences, Faculty of Veterinary Science, University of Pretoria, Pretoria, Gauteng, 0110, South Africa; 2 Centre for Veterinary Wildlife Studies, Faculty of Veterinary Sciences, University of Pretoria, Pretoria, Gauteng, 0110, South Africa; 3 Wellcome Genome Campus, Wellcome Sanger Institute, Hinxton, England, CB10, UK

**Keywords:** Schwannoma, melanocytoma, melanoma, melanin, neoplasm, verocay body, dog

## Abstract

Schwannoma is a nerve sheath tumour arising from differentiated Schwann cells, and melanocytic schwannoma (MS) is a rare variant where the Schwan cells produce melanin pigment. MS is typically associated with spinal nerve roots and there have been only ~20 reports of cutaneous or subcutaneous MS to-date in humans. In canines, there have only been two reports of MS, both associated with spinal root nerves. In this report, we describe a 7-year-old Weimaraner cross breed dog that presented with two pigmented lesions on the eyelids. The lesions were surgically removed and histological analysis revealed well-circumscribed, non-encapsulated, expansile, neoplasms that were displacing most of the dermis and adnexa. The first lesion was composed of spindloid cells arranged in short interlacing streams with large amounts of pale eosinophilic cytoplasm that sometimes contained fine melanin granules. In areas there were spindle cells arranged in verocay bodies which led to a diagnosis of MS. In contrast, the second lesion was composed of polygonal cells arranged in thick sheets with large amounts of pale eosinophilic cytoplasm that sometimes contained fine melanin granules. The diagnosis was melanocytoma (which is one of the macroscopic differential diagnoses for MS). Whilst melanocytoma is a commonly occurring cutaneous lesion in canines and surgical removal is considered curative, due to little being known about MS in dogs, the outcome remained guarded, as MS in humans has an unpredictable nature, and recurrence and metastasis have been reported.

## Background

Schwannoma is a nerve sheath tumour arising from differentiated Schwann cells and in adult humans it is the most common type of benign peripheral nerve tumour. Although typically associated with spinal nerve roots, schwannomas can occur as a primary neoplasm in the skin, soft tissue and visceral organs ^1^. Melanocytic schwannoma (MS), or melanotic schwannoma, is a rare variant of nerve sheath tumour composed of Schwann cells that produce melanin ^2^. MS accounts for <1% of primary peripheral nerve sheath tumours in humans ^1^ and only ~200 cases of MS have been described in the literature, with only ~20 of these cases being cutaneous and subcutaneous MS ^3– 6^, highlighting the rarity of MS at this tissue site. In dogs, there have only been two cases of MS described in the literature; a single study of two dogs from nearly 40 years ago (a 2 year-old mixed breed male and Doberman pinscher female) ^7^. Both dogs presented with a 2 week period of progressive uncoordination/leg weakness, and examination revealed a lesion at the T12 region of the male and cauda equina of the female (leading to the owners’ decision to euthanise) ^7^. Thus our case study is the first report of cutaneous MS in a dog, with a concomitant melanocytoma.

## Case report

A 33kg 7-year-old female spayed Weimaraner cross breed from Pretoria, South Africa, was presented to a specialist veterinary ophthalmologist for consultation about a large pigmented growth on her left lower eye lid, amongst another smaller pigmented eyelid mass and two larger soft subcutaneous masses in the right axilla and the left ventral abdomen respectively. The dog had not visited the Onderstepoort Veterinary Academic Hospital prior to this presentation, and no other abnormalities were detected with her eyes, although some nuclear sclerosis was noted, which is common in older dogs. A blood smear found red blood cells, white blood cells and platelets to all be present with no abnormalities or parasites detected. Fine needle aspirates were performed on both soft subcutaneous masses and only adipocytes were seen in the cytology smears. The owner elected for all four lesions to undergo surgical resection under general anaesthesia and the masses were placed in 10% buffered formalin for histopathological analysis at the Faculty of Veterinary Science, University of Pretoria. The dog was discharged back to the owner the next day and returned 14 days later to Outpatients for removal of the sutures.

**Figure 1.**
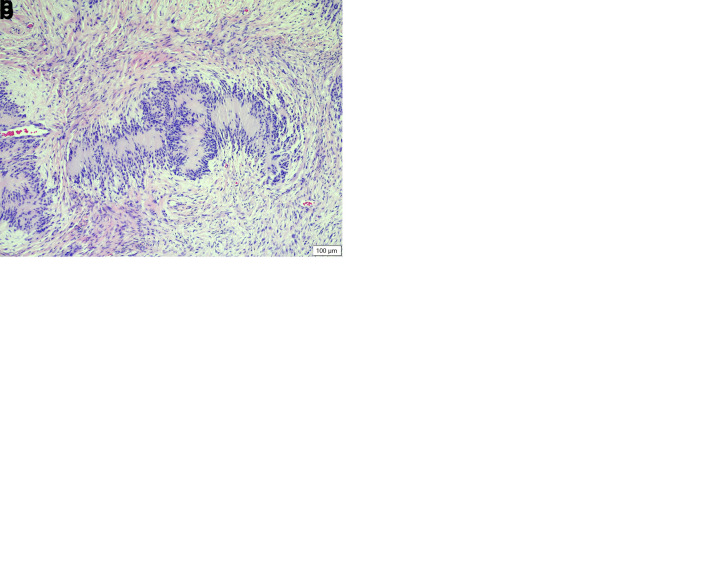
Histopathology of the melanocytic schwannoma (neoplasm A). ( **a**) Low magnification revealing the non-encapsulated, expansile, cell dense proliferation of neoplastic spindloid cells displacing the dermis and adnexa. Also note the verocay bodies present within this cellular proliferation (HE stain, 40× magnification). ( **b**) High magnification revealing the cellular morphology of the pigmented neoplastic cells (HE stain, 400× magnification). ( **c**) Presence of distinct verocay bodies within the neoplastic proliferation (HE stain, 100× magnification).

**Figure 2.**
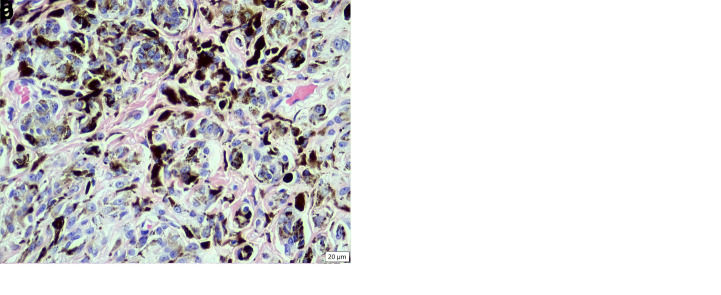
Histopathology of the melanocytoma (neoplasm B). ( **a**) Low magnification revealing the pedunculated, non-encapsulated, expansile, cell dense proliferation of neoplastic pigmented spindloid cells displacing the dermis and adnexa (HE stain, 40× magnification). ( **b**) High magnification revealing the cellular morphology of the highly pigmented neoplastic cells (HE stain, 400× magnification).

The formalin-fixed tissues were embedded in paraffin wax, sectioned and stained with haematoxylin and eosin. Both eyelid growths (A – larger, and B – smaller) were pedunculated, firm black dermal masses that histologically presented as well-circumscribed, non-encapsulated, expansile, cell dense neoplasms with clean margins, that were displacing most of the dermis and adnexa, with the overlying epidermis being mildly hyperplastic and characterised by acanthosis and elongated rete pegs ( Figure 1a and Figure 2a). Neoplasm A was composed of spindloid cells arranged in short interlacing streams and supported by a small amount of fibrovascular stroma ( Figure 1b). The cells had variably indistinct cell borders, large amounts of pale eosinophilic cytoplasm that sometimes contained fine melanin granules, as well as oval to elongated basophilic nuclei with finely stippled chromatin, and variably distinct central nucleoli. In areas there were spindle cells arranged in verocay bodies ( Figure 1c). There was moderate anisokaryosis and anisocytosis, with only one mitotic figure seen. In the adjacent dermis, there was multifocal mild to moderate ectasia of sweat glands, a moderate multifocal inflammatory cell infiltrate (predominately neutrophils and macrophages) around the adnexal structures and a moderate number of randomly scattered melanomacrophages. The diagnosis was melanotic schwannoma.

Neoplasm B was composed of polygonal cells arranged in thick sheets supported by a moderate amount of fibrovascular stroma ( Figure 2b). The cells had indistinct cell borders and large amounts of pale eosinophilic cytoplasm that sometimes contained fine melanin granules, as well as round to oval nuclei with coarse sparse chromatin and a variably distinct central single nucleolus. There was moderate anisokaryosis and anisocytosis, and no mitotic figures were seen. There were single or clusters of neoplastic cells scattered in fairly large numbers along the epidermal-dermal junction and rarely in the epidermis, and multifocally interspersed with moderate numbers of melanomacrophages. The diagnosis was melanocytoma.

The soft subcutaneous masses, two smooth-surfaced masses measuring approximately 20 × 15 × 10mm and 35 × 20 × 20mm respectively, were composed of mature/well-differentiated adipocytes with no spindle cell proliferation and minimal fibrovascular stroma, consistent with that seen in the cytology smears ( Figure 1c). The diagnosis was lipoma, which is a benign tumour that is very common in dogs.

A year after removal of the masses, the owner was contacted and confirmed there was no regrowth.

## Discussion

The 2007 World Health Organization (WHO) classification of human peripheral nerve tumours (PNTs) has been adapted for veterinary use due to the gross and histological similarities between these tumours in humans and dogs ^8^. There are four major subtypes of PNT, namely schwannoma, neurofibroma, perineuroma and malignant peripheral nerve sheath tumour (PNST). In the dog, the most common subtype of PNT is schwannoma, which is a benign encapsulated nerve sheath tumour composed of well-differentiated neoplastic Schwann cells ^8^. Canine schwannomas are typically found intradurally within spinal nerve roots or extradurally in the brachial plexus and consist of relatively densely packed cells with ovoid to elongated fusiform shapes and eosinophilic cytoplasm with indistinct borders, supported by a variably dense collagen matrix ^8^. The tumours are composed of dense cellular sheets arranged in patterns of interwoven bundles, concentric whorls or streams; in humans this dense cellular form is classified as the Antoni type A pattern ^9^ and it is the most common type seen in the dog ^8^. In contrast to humans where verocay bodies (formed by stacked parallel rows of palisading nuclei separated by anuclear zones) are a common occurrence and pathognomic ^10^, verocay bodies are extremely rare in dogs ^8^.

MS is one of the rare variants of schwannoma, with the cells displaying ultrastructural features of Schwann cells but also possessing melanosomes. They are typically found in the gastrointestinal tract and paraspinal sympathetic chain, with only ~20 reports of cutaneous and subcutaneous MS in humans to-date ^3– 6^ and none in animals. We present here the first report of cutaneous MS in an animal, specifically a dog that presented with two cutaneous pigmented lesions, a melanocytoma and a MS. Melanocytoma and malignant melanoma are differential diagnoses for MS and it can be difficult to differentiate between these tumour types as they generally express S-100, due to their shared neural crest origin, and one or more melanosomal markers ^1, 3^. However, melanocytomas and melanomas do not display evidence of schwannian differentiation, specifically verocay bodies, and together with the proliferation of pigmented spindle cells seen in this case, the diagnosis of MS was made. The presence of verocay bodies have been previously reported in some cases of canine schwannoma ^10^ but not others ^11^.

Conventional schwannoma is typically an encapsulated, benign tumour. In contrast, whilst MS is generally considered a benign lesion, it is typically a circumscribed yet unencapsulated tumour, which may reflect its potentially more aggressive nature, consistent with its unpredictable clinical course. Indeed, the benign nature of this tumour has come from case studies with only a short-term follow up, as there have been reports of recurrence (15–35%,) and metastasis (26–44%) in patients with long-term follow up (3–7 years) ^12– 16^. In humans, MS has been reported to metastases to the following sites, lung and pleura, mediastinum, diaphragm, pericardium, endocardium, bone, liver, and spleen ^17^. In addition, since clinicopathologic evaluation is a poor predictor of the biologic behavior of MS ^13^, appropriate long-term follow-up has been recommended as it may recur or metastasize even in the absence of overt malignant features.

In contrast to MS, melanocytoma is a benign neoplasm arising from the melanocytes in the epidermis, dermis or adnexa, and is common in dogs ^18^. In dogs, melanocytomas have a predilection for the eyelids. The majority of canine melanocytomas are slow-growing and amenable to surgical excision, with ~90% of dogs with cutaneous melanocytomas reported to be alive after 2 years (in comparison with dogs with cutaneous melanoma, in which 50% lived less than 7 months after diagnosis ^18^). Thus whilst both the excised lesions in this case showed clear margins, which lessens the chance of recurrence, the overall prognosis remains guarded and close follow up is recommended, due to the paucity of information of MS in canines and the unpredictable nature of this tumour type in humans.

In conclusion, our case report adds to the limited knowledge of this tumour type that is rare in both humans and animals, especially a cutaneous presentation. The rarity of this tumour type highlights the benefits of a ‘One Medicine/One Health’ approach ^19^, where human and veterinary medical experts combine their experience/knowledge and what we learn in one species can be used to benefit the other.

## Consent

Written informed owner consent has been obtained from the owner of the patient to publish this paper. The Research Ethics Committee of the Faculty of Veterinary Science at the University of Pretoria approved this case report (REC160-22).

## Data Availability

All data underlying the results are available as part of the article and no additional source data are required. Figshare: CARE checklist for ‘Case Report: Cutaneous melanocytic schwannoma with concomitant melanocytoma in a canine’. https://doi.org/10.6084/m9.figshare.23896725 Data are available under the terms of the Creative Commons Attribution 4.0 International license (CC-BY 4.0).
