# Supplementary material for: Case Report: Cutaneous melanocytic schwannoma with concomitant melanocytoma in a canine
Source: Wellcome Open Res. 2024 Feb 26;8:364. Originally published 2023 Aug 24. [Version 2] doi: 10.12688/wellcomeopenres.19694.2 (PMC11190644; doi:10.12688/wellcomeopenres.19694.2)
Supplement: Peer review history

# Reviewer report: Reviewer response for version 2

**DOI:** 10.21956/wellcomeopenres.23197.r85470

**Published:** 2024-07-03

**Authors:** Rebecca C Smedley

**Affiliations:** 1 Michigan State University, Michigan, USA

Thank you for describing this tumor with an uncommon cellular pattern. It highlights the need to better characterize pigmented tumors with neural patterns. I agree with the reviewer who indicated that immunohistochemistry is necessary to make this diagnosis, however. Electron microscopy would also be nice. 

 I frequently see cutaneous melanocytomas that have a neural appearance. I would argue that it may be possible for melanocytic neoplasms to form Verocay bodies in some occasions. Melanocytic neoplasms have a wide range of patterns, and I don't think you can use pattern alone to determine cell type. There is a case in the human literature of a purported dermatofibroma that exhibited Verocay bodies for example ^1^. Another paper described Verocay bodies in a malignant melanoma ^2^. A third human paper stated: "Recent evidence shows that a subset of melanocytes develop from schwannian precursors that detach from developing nerves during normal development ^3^. This provides a potential biological mechanism for the observation that melanocytic nevi often have areas of schwannian differentiation. This observation suggests that a tumor could potentially show differentiation that parallels this developmental pathway and exhibit both schwannian and melanocytic attributes yet lack the full characteristics of melanocytes, including a lack of ability to produce melanin." ^3^.

 Some papers state that peripheral nerves need to be present in the section to diagnosis a schwannoma. Thus, I think differentiation between a pigmented schwannoma and a melanocytic neoplasm is much muddier than simply looking at the pattern/arrangement of the cells. IHC may still not be definitive, but I would like to see Melan-a, PNL-2, TRP-2, and laminin at least.

 If there are neoplastic melanocytes within the overlying epithelium, which I wondered about in the provided image but could not tell for sure at that magnification, I would diagnose this mass as a melanocytic neoplasm and not a schwannoma. It was also not clear if both masses were from the same eye, the same eyelid, or different eyelids.

Are enough details provided of any physical examination and diagnostic tests, treatment given and outcomes?

Yes

Is the case presented with sufficient detail to be useful for other practitioners?

No

Is sufficient discussion included of the importance of the findings and their relevance to future understanding of disease processes, diagnosis or treatment?

Partly

Is the background of the case’s history and progression described in sufficient detail?

Yes

Reviewer Expertise:

Tumor pathology, melanomas in particular

I confirm that I have read this submission and believe that I have an appropriate level of expertise to state that I do not consider it to be of an acceptable scientific standard, for reasons outlined above.

## References

[1] : Dermatofibroma with Verocay Body-Type Palisading Features and a Brief Discussion on Potential Schwannoma Mimickers of the Skin. *Turk Patoloji Derg* .2023;39(3) : 10.5146/tjpath.2023.01602 218-220 10.5146/tjpath.2023.01602 37032629 PMC10521201

[2] : Malignant melanoma simulating schwannian differentiation. *Am J Dermatopathol* .1985;7 Suppl: 10.1097/00000372-198501001-00032 171-6 10.1097/00000372-198501001-00032 3940081

[3] : Plexiform melanocytic schwannoma: a mimic of melanoma. *J Cutan Pathol* .2012;39(5) : 10.1111/j.1600-0560.2011.01856.x 521-5 10.1111/j.1600-0560.2011.01856.x 22416678

# Reviewer report: Reviewer response for version 2

**DOI:** 10.21956/wellcomeopenres.23197.r85464

**Published:** 2024-06-20

**Authors:** Maria Teresa Mandara

**Affiliations:** 1 University of Perugia, Perugia, Italy

- The paper describes an interesting differential diagnosis exercise concerning a cutaneous melanocytic schwannoma vs cutaneous melanocytoma for a rare neoplastic lesion such as the melanocytic schwannoma is in the dog. Histologically both the tumors can express mesenchymal and epithelial patterns since they share similar embryonal origin from the neural crests. This makes very difficult to base diagnosis just on morphological findings, especially in the cutaneous site. Verocay bodies are histological patterns strongly suggesting the diagnosis of schwannoma. So, if they can be sufficient to differentiate melanocytoma from MS (lesion 1), not the same can be assumed in the second lesion which, in the absence of similar pattern, needs to be differentiated from MS. A great support for final diagnosis could be provided from immunohistochemistry referred to laminin, EMA, etc. Actually, we know that both the tumors share similar markers so that discussion about this point needs to be included.
- Actually, since Verocay bodies are rarely expressed by the canine schwannoma, they can’t be the base for the current diagnosis of schwannoma. Looking at Fig.1b and Fig.2b it is difficult to differentiate one tumor from the other. The first one could be melanocytic tumor having a mesenchymal pattern and the latter a schwannoma mimicking epithelial features. Starting from this point, immunolabelling can really add useful information in the diagnosis of cutaneous melanocytic schwannoma, maybe not. What your opinion? Please, discuss this point.
- As an option to immunolabelling, the author could try to perform electron microscopy form formalin fixed samples.

Backgroound

- *in adult humans it is the most common type of benign peripheral nerve tumour *(address to reference)
- *In dogs, there have only been two cases of MS described in the literature; a single study of two dogs from nearly 40 years ago (a 2 year-old mixed breed male and Doberman pinscher female) * A further study should worth adding. (Case report: MRI and CT imaging features of a melanocytic tumour affecting a cervical vertebra in an adult dog, and review of differential diagnosis for T1W-hyperintense lesions. Elli Elizabeth Michaelidou, ^^et al. 2024)

Case Report

- What the weight of the two soft cutaneous masses for the purpose of this study?
- Fig. 1c does not correspond to the text. Please, verify this picture.

Discussion

*together with the proliferation of pigmented spindle cells seen in this case, the diagnosis of MS was made.*

- If Verocay bodies can be sufficient to differentiate melanocytoma from MS (lesion 1), not the same can occur for the second lesion which, in the absence of similar pattern, should be differentiated from MS based on immunohistochemistry or electron microscopy.

Are enough details provided of any physical examination and diagnostic tests, treatment given and outcomes?

Partly

Is the case presented with sufficient detail to be useful for other practitioners?

Yes

Is sufficient discussion included of the importance of the findings and their relevance to future understanding of disease processes, diagnosis or treatment?

Partly

Is the background of the case’s history and progression described in sufficient detail?

Yes

Reviewer Expertise:

Neuropathology

I confirm that I have read this submission and believe that I have an appropriate level of expertise to confirm that it is of an acceptable scientific standard, however I have significant reservations, as outlined above.

# Reviewer report: Reviewer response for version 2

**DOI:** 10.21956/wellcomeopenres.23197.r75440

**Published:** 2024-03-07

**Authors:** Tatsuya Deguchi

**Affiliations:** 1 Faculty of Veterinary Medicine, Veterinary Teaching Hospital, Hokkaido University & Department of Companion Animal Clinical Sciences, Companion Animal Internal Medicine, School of Veterinary Medicine, Rakuno Gakuen University, Ebetsu, Japan

I think this revised report reflects my comments.

Are enough details provided of any physical examination and diagnostic tests, treatment given and outcomes?

Partly

Is the case presented with sufficient detail to be useful for other practitioners?

Yes

Is sufficient discussion included of the importance of the findings and their relevance to future understanding of disease processes, diagnosis or treatment?

Yes

Is the background of the case’s history and progression described in sufficient detail?

Yes

Reviewer Expertise:

Small animal, dogs, radiation therapy, tumor

I confirm that I have read this submission and believe that I have an appropriate level of expertise to confirm that it is of an acceptable scientific standard.

# Reviewer report: Reviewer response for version 1

**DOI:** 10.21956/wellcomeopenres.21815.r70703

**Published:** 2024-03-21

**Authors:** Volkan Ipek

**Affiliations:** 1 Faculty of Veterinary Medicine, Department of Pathology, Burdur Mehmet Akif Ersoy University, Burdur, Turkey

The article presents a detailed and insightful case study of a rare melanotic schwannoma (MS) in a dog, highlighting the challenges and nuances in diagnosing such uncommon tumors. One of the key strengths of the paper is its thorough exploration of the histopathological characteristics of MS, particularly the identification of specific features such as Verocay bodies, which play a critical role in differentiating MS from other melanocytic tumors like melanocytomas and melanomas.

 However, I believe that the article could further enrich its contribution to the literature by incorporating the use of basic immunohistochemical markers, including nerve and melanocytic markers. Although the histopathological characteristics are sufficient for diagnosing this subtype, immunohistochemistry in such a rare tumor could provide a more comprehensive understanding of its cellular composition and behavior. The use of such markers could potentially offer more definitive insights, especially in distinguishing MS that does not show the characteristic histological findings of a schwannoma.

 Overall, while the paper makes a significant contribution to the field of veterinary oncology by documenting a rare case of cutaneous MS in a dog, its impact could be further enhanced with a deeper exploration of immunohistochemical characteristics. This addition would not only strengthen the diagnostic process but also provide valuable insights into the tumor's pathology, potentially benefiting future research and clinical practices in the field.

Are enough details provided of any physical examination and diagnostic tests, treatment given and outcomes?

Partly

Is the case presented with sufficient detail to be useful for other practitioners?

Yes

Is sufficient discussion included of the importance of the findings and their relevance to future understanding of disease processes, diagnosis or treatment?

Partly

Is the background of the case’s history and progression described in sufficient detail?

Yes

Reviewer Expertise:

Dogs, immunohistochemistry, tumor diagnosis

I confirm that I have read this submission and believe that I have an appropriate level of expertise to confirm that it is of an acceptable scientific standard, however I have significant reservations, as outlined above.

# Response

**Published:** 2024-03-30

**Authors:** Olwam Monakali

**Affiliations:** Paraclinical Sciences, Faculty of Vet Sciences, University of Pretoria, Pretoria, Gauteng, South Africa

Thank you for affording us the opportunity to revise our manuscript entitled “Case Report: Cutaneous melanocytic schwannoma with concomitant melanocytoma in a canine”. Please find our comments below.  

*Reviewer 2 The article presents a detailed and insightful case study of a rare melanotic schwannoma (MS) in a dog, highlighting the challenges and nuances in diagnosing such uncommon tumors. One of the key strengths of the paper is its thorough exploration of the histopathological characteristics of MS, particularly the identification of specific features such as Verocay bodies, which play a critical role in differentiating MS from other melanocytic tumors like melanocytomas and melanomas.*

 We thank the reviewer for their appreciation of our case study, specifically the detailed histopathological characteristics.

*However, I believe that the article could further enrich its contribution to the literature by incorporating the use of basic immunohistochemical markers, including nerve and melanocytic markers. Although the histopathological characteristics are sufficient for diagnosing this subtype, immunohistochemistry in such a rare tumor could provide a more comprehensive understanding of its cellular composition and behavior. The use of such markers could potentially offer more definitive insights, especially in distinguishing MS that does not show the characteristic histological findings of a schwannoma.*

 Many studies in human pathology have demonstrated that MS typically shows immunohistochemical positivity for HMB-45, S100, and vimentin [1-5], and schwannomas typically show immunohistochemical positivity for CD56, S100, and vimentin [6-9]. Thus, we would want to use the neurogenic marker CD56, which is typically positive in schwannomas and negative in MS, and the melanocytic marker HMB45, which his typically negative in schwannomas and positive in MS. However, we do not have these antibodies available in our diagnostic facility. We have S100 and vimentin antibodies, but as they stain positive for both schwannomas and MS (as well as a variety of other tumour types), it would not add any additional information.   We understand that performing IHC would certainly be interesting. However, since this was a diagnostic case report, we feel that performing IHC is beyond the scope of our report, as the reviewer themselves acknowledge that “the histopathological characteristics are sufficient for diagnosing this subtype”.    References:  1. Jensen OA et al., 1990. PMID: 2206515 2. Myers JL et al. 1990. PMID: 1689947 3. Zhang HY, et al. 2005.  PMID: 16157048. 4. Kang, YE, et al., 2018. PMID: 29132201 5. Soyland, DJ. Et al. 2021. PMID: 33948334 6. Taconet, S. et al., 2014. PMID: 25310741 7. Zhou, J. et al., 2015, PMID: 26191302 8. Agaimy, A & Wunsch PH, 2017. PMID: 17923471 9. Jaiswal P, et al., 2023. PMID: 37485115

*Overall, while the paper makes a significant contribution to the field of veterinary oncology by documenting a rare case of cutaneous MS in a dog, its impact could be further enhanced with a deeper exploration of immunohistochemical characteristics. This addition would not only strengthen the diagnostic process but also provide valuable insights into the tumor's pathology, potentially benefiting future research and clinical practices in the field.*

 We are delighted that the reviewer feels our case report makes a “significant contribution to the field of veterinary oncology” and hope they can understand our rationale for not performing the additional immunohistochemical characterisation. We hope our case report is now suitable for indexing.     

 Yours Sincerely,   Olwam Monakali.

# Reviewer report: Reviewer response for version 1

**DOI:** 10.21956/wellcomeopenres.21815.r68368

**Published:** 2023-10-30

**Authors:** Tatsuya Deguchi

**Affiliations:** 1 Faculty of Veterinary Medicine, Veterinary Teaching Hospital, Hokkaido University & Department of Companion Animal Clinical Sciences, Companion Animal Internal Medicine, School of Veterinary Medicine, Rakuno Gakuen University, Ebetsu, Japan

This case report is well-designed. Please make the following revisions:

1. Include macroscopic findings and photographs taken during the surgery.
2. Provide detailed surgical findings such as surgical margins.
3. Regarding the sentence "A year after removal of the masses, the owner was contacted and confirmed there was no regrowth," include information about evaluations for metastasis, such as chest X-rays, and record the subsequent follow-up related to metastasis.
4. Add details about the site of metastasis in human tumors.
5. Discuss the pathological findings to support the benign behavior of canine MS.

Are enough details provided of any physical examination and diagnostic tests, treatment given and outcomes?

Partly

Is the case presented with sufficient detail to be useful for other practitioners?

Yes

Is sufficient discussion included of the importance of the findings and their relevance to future understanding of disease processes, diagnosis or treatment?

Yes

Is the background of the case’s history and progression described in sufficient detail?

Yes

Reviewer Expertise:

Small animal, dogs, radiation therapy, tumor

I confirm that I have read this submission and believe that I have an appropriate level of expertise to confirm that it is of an acceptable scientific standard, however I have significant reservations, as outlined above.

# Response

**Published:** 2024-01-26

**Authors:** Olwam Monakali

**Affiliations:** Paraclinical Sciences, Faculty of Vet Sciences, University of Pretoria, Pretoria, Gauteng, South Africa

Firstly, thank you for the feedback Tatsuya Deguchi.

1. Unfortunately, the surgeon responsible for the case has seen left our University which has made it difficult to for us to gather macro-surgical information (pictures).
2. Microscopically, the margins were clean and this will be included in the report.
3. Shortcoming: There was no diagnostic long term follow up. The client feedback served sufficient for the clinical clinicians.
4. According to Pathology of Melanotic Schwannoma article by Borislav A. Alexiev; Pauline M. Chou; Lawrence J. Jennings. The following have been reported as sites for metastasis are lungs and pleura mediastinum, diaphragm, pericardium, endocardium, bone, liver, and spleen.
5. Microscopically, there cellular and morphological characteristics of the tumour were indicative of a benign. Although there was moderate anisokaryosis and anisocytosis, there is was only one mitotic figure seen in the tumour sections examined. As discussed in the case report, the tumours may have metastatic potential, but there is limited animal literature on this, as such the prognosis was guarded.
